# Research with cancer registry data in radiation oncology—current possibilities and perspectives: A narrative review

**DOI:** 10.1007/s00066-025-02486-w

**Published:** 2025-11-10

**Authors:** Jörg Andreas Müller, Ahmed Bedir, Alexander Katalinic, Florian Oesterling, Aleksandra Graw, Alexander Kluttig, Stefanie Corradini, Dirk Vordermark, Daniel Medenwald

**Affiliations:** 1 Department of Radiation Oncology, University Hospital Halle (Saale), Ernst-Grube-Str. 40, 06120 Halle (Saale), Germany; 2 University Clinic for Radiation Therapy, University Hospital Magdeburg A. ö. R, Magdeburg, Germany; 3 Institute of Social Medicine and Epidemiology, University of Lübeck, Ratzeburger Allee 160, 23562 Lübeck, Germany; 4 Cancer Registry North Rhine-Westphalia, 44801 Bochum, Germany; 5 Clinical Cancer Registry Saxony-Anhalt (Klinische Krebsregister Sachsen-Anhalt GmbH), Doctor-Eisenbart-Ring 2, 39120 Magdeburg, Germany; 6 Department of Radiation Oncology, University Hospital, LMU Munich, Marchioninistr. 15, 81377 Munich, Germany

**Keywords:** Cancer registry, Radiation oncology, Data completeness and quality, Real-world evidence, Methodological challenges

## Abstract

**Clinical background:**

Cancer registry data are an essential resource for population-based oncology research and quality assurance in Germany. With the revision of the Federal Cancer Registry Data Act in 2021, for the first time, a legal basis was created for the development of a nationwide clinical cancer dataset. This registry offers new opportunities for research, particularly in radiation oncology.

**Objective:**

This review aims to provide an overview of the current possibilities of and challenges to using German cancer registry data in radiation oncology, with a focus on data structure, missing data, access procedures, and methodological aspects for scientific research.

**Methods:**

We examined legal frameworks, data access procedures, and the structure of the nationwide basic oncology dataset (oBDS), particularly the components relevant to radiotherapy (RT). The completeness and comprehensiveness of RT data across federal states were assessed using national registry data from the German Center for Cancer Registry Data (ZfKD) for the years 2020–2022.

**Results:**

The cancer registries provide structured data on RT through the oBDS, including treatment intent, technique, target area, and side effects. However, significant variability in terms of completeness and reporting standards persists among federal states. Missing data rates remain high in some regions, particularly for key RT parameters such as treatment technique, the relationship to surgery, and boost application. Methodological challenges for scientific use include handling missing data, confounding, immortal time bias, and exposure misclassification.

**Conclusion:**

Despite existing limitations, cancer registry data in Germany represent a valuable resource for real-world research in radiation oncology. They allow for large-scale population-based studies beyond the constraints of clinical trials. With improved data harmonization, methodological rigor, and future data linkages (e.g., to health insurance data), registry-based studies can contribute significantly to evidence-based decision-making and quality improvement in radiation oncology.

## Clinical background

The cancer registries of the German federal states fulfill legally defined tasks in the field of public health, such as supporting health reporting and cancer research, as well as actively participating in quality assurance for oncological care and early cancer detection [1–3]. Cancer registry analyses play a leading role in mapping care structures and processes, particularly in oncological care research [4]. To name just one important field of research, care deficits in the sense of deviations from guideline-based treatment can be systematically identified and addressed in this context [2]. It is well known from previous work that guideline-adherent treatment can significantly improve the survival of cancer patients with various entities [5–7].

The revision of the Federal Cancer Registry Data Act in 2021 laid, for the first time, the legal groundwork for establishing a nationwide clinical cancer dataset in Germany [8]. At the same time, the establishment of state-specific clinical cancer registries based on the Cancer Early Detection and Registration Act § 65c SGBV was completed in 2020 [8].

Due to the regular integration of radiation therapy into multidisciplinary treatment of oncological diseases, the use of cancer registry data for clinical cancer research is subject to various special considerations that researchers should take into account at an early stage. At the same time, however, these data sources allow for an evaluation of treatment options outside the limited setting of clinical trials [9, 10].

This paper aims to provide an overview of the possibilities for scientific use of cancer registry data in the field of radiation oncology. On the one hand, practical aspects such as data applications will be examined and a description of the structure and missing data of the radiation oncology data in the nationwide dataset provided. On the other hand, methodological issues specific to the use of cancer registry data will be addressed.

## Methods

### Data request process

The provision of clinical cancer registry data for health services research is a central task of the German Center for Cancer Registry Data (ZfKD) and the state cancer registries. Researchers can request data from both the ZfKD and the state cancer registries.

The ZfKD provides epidemiological and limited clinical datasets (the current observation period extends from the diagnosis year 2020 to 2023; restricted dataset) at the federal level, which are suitable for projects with broad data requirements. These restricted datasets exclude detailed clinical data such as treatment specifics (e.g., radiation dose, chemotherapy regimens), side effects, and patient contact information. Applications are submitted directly to the ZfKD, after which they are reviewed by a scientific committee. Further information on the procedure and the scope of data available can be found at www.krebsdaten.de.

For projects that require additional information beyond the ZfKD data, such as cohort comparisons, research approaches that require patient contact, or studies such as survival analyses (with follow-up varying depending on the respective registry), the data can be requested from both the ZfKD and the state cancer registries. Vital status data are available at the ZfKD as well, although follow-up procedures may vary slightly between registries. The ZfKD can provide the necessary background information, and the data are generally comparable to those from the state cancer registries, with the exception that the latter may be slightly more up to date. It is also possible to evaluate the significance of treatment location and the type of facility (e.g., outpatient institution or hospital) using data from the state cancer registries.

Cancer registry data offer various categories of data, which are usable for scientific purposes. This includes aggregated data consisting of summarized individual data based on parameters such as age, sex, year of diagnosis, or region. Aggregated data are easily accessible and go through a simplified application process. For more detailed analyses, researchers can request individual case data, which is usually provided in anonymized form. In addition, researchers can link cancer registry data with existing cohort studies to compare cancer outcomes. This linkage allows for the analysis of cancer-related outcomes in external cohorts and requires a common identifier for proper matching. If the study involves linking with external data, informed consent from study participants is required. It is important to note that cohort studies may also be epidemiological studies with different legal requirements, especially when no intervention is involved, and, therefore, the legal framework may differ from that of clinical trials.

The process for requesting cancer registry data is similar for all cancer registries. However, there are some special aspects under state law that scientists must take into account. Ongoing harmonization of the application procedure between cancer registries has already led to a standardized application form (https://plattform65c.de/wp-content/uploads/2025/04/2025-04-01-Antrag-auf-Nutzung-von-Krebsregisterdaten-final-geschuetzt-DE.docx). This application form must describe the research objectives, data requirements, target population, evaluation methodology, and intended duration of use. In general, the principle of data minimization applies, so that only variables and cases that are necessary to answer the research question are provided.

In all cancer registries, applications for use are reviewed by both an internal and an external committee. For example, in the Saxony-Anhalt Cancer Registry, incoming applications are first evaluated by an internal committee consisting of the management, the head of the evaluation unit, and the head of data management. The application is reviewed according to various criteria, including scientific quality, legal and ethical compliance, and the feasibility of providing the requested data. Any ambiguities are discussed directly with the applicants. The committee ensures that the requested data are consistent with the objectives of the project and that the principle of data minimization is pursued. After review by the internal committee, the application is forwarded to the scientific advisory board for final approval. This phase includes a 4-week objection period during which advisory board members can raise concerns. If no objections are raised, the approval process is completed. For applications involving personally identifiable data, such as contacting cancer patients or cohort matching, a detailed scientific study protocol, approval from an ethics committee, and, if necessary, additional approvals (e.g., from data protection authorities or relevant ministries) are usually required.

After approval, the requested data are prepared for secure transfer to the researcher. The process may include additional anonymization or pseudonymization steps to ensure that re-identification of individuals is unlikely. Using secure methods, the registry transfers the data to the researcher. The user is obliged to follow strict protocols to maintain the confidentiality and integrity of the data throughout the research project. At the same time, a usage agreement is concluded, which stipulates, among other things, that attempts at re-identification are prohibited and that the data may only be used for the purpose stated in the application and must be deleted at the end of the project. Furthermore, there is generally a reporting obligation to the data providing cancer registry, whereby the submission of publications resulting from the project is sufficient.

For research projects that include data from several federal states and/or need more detailed information than the ZfKD dataset, researchers currently still have to submit separate applications to each regional cancer registry of the respective federal state. This means that different legal requirements must be met, and the processing time may vary depending on the federal state. However, efforts are underway to simplify the application process nationwide so that data from several registries can be requested with a single application in the future.

### The nationwide basic oncology dataset

The Cancer Registry and Early Detection Act (KFRG, 2013) introduced nationwide clinical cancer registration and established a federal, binding basic oncology dataset (oBDS) for documenting cancer and its treatment. The German Cancer Registry (DKR/formerly GEKID) and the Working Group of German Tumor Centers (ADT) have the legal responsibility under Section 65c of the German Social Code (SGB V) to define and maintain the oBDS with its specific modules in cooperation with the Section 65c Platform (coordination group of the state cancer registries). Version 3.0 was released in October 2021 and will be in widespread use by mid-2024.

The dataset comprises around 130 data parameters and is divided into information on the person affected, the tumor disease, the various forms of treatment, and follow-up (Table 1). Up to 15 different pieces of information may be required to document radiation therapy.

**Table 1:** The basic oncological dataset for cancer registration

**Basic dataset**	Data fields (approx. 130)	
Personal data	Health insurance number, names, address, date of birth, gender	
Information on the reporting center	Institution, name, address	
Tumor diagnosis	ICD-10, ICD‑O localization, date of diagnosis, confirmation of diagnosis, site localization	
Histology	ICD‑O morphology, grading, number of lymph nodes examined and affected	
Tumor classification	TNM and other classification	
Genetic variant	Type	
Residual status	After surgery and overall assessment	
Performance status	ECOG (at diagnosis and follow-up)	
Surgery	Date, intention, OPS coding, complications	
*Radiotherapy*	*Intention*	*Curative, palliative, local curative for oligometastasis, other*
	*Relationship to surgery*	*Not related to surgical treatment, adjuvant, neoadjuvant, intraoperative, additive, other*
	*Target area*	*According to target area key, with page reference if applicable*
	*Start and end date*	*Exact date*
	*Type of application*	*According to list of possible techniques*
	*Type of radiation*	*Applied radiation type according to list*
	*Total dose*	*Total dose in target area including boost*
	*Boost*	*Information on whether boost was applied (list)*
	*End of RT reason*	*Regular end, target dose reached interrupted for >* *3 calendar days, discontinued due to side effects, discontinued due to progression, discontinued for other reasons, patient refused further therapy, patient deceased*
	*Side effects*	*According to CTCAE criteria, type of side effects*
Systemic therapy	Date, intention, position regarding surgery, type of therapy (including active surveillance), protocol, substance, start, end	
Tumor board	Date, type	
Follow-up	Date, overall assessment of tumor, lymph nodes, and metastases	
Death	Date, cause	
**Organ-specific modules**	Specific content; the official names of the additional modules can be found at https://plattform65c.atlassian.net/wiki/spaces/Dokumentat/pages/86442141/Zusatzmodule	
Breast cancer	10 variables	
Prostate cancer	10 variables	
Colorectal cancer	12 variables	
Melanoma of the skin	4 variables	

The characteristics relating to radiation therapy are presented in more detail, while the other fields and modules (oDBS 3.0) are simplified. A detailed description of the individual data fields and their characteristics can be found at https://basisdatensatz.de/basisdatensatz

*CTCAE* Common Terminology Criteria for Adverse Events*. ECOG* Eastern Cooperative Oncology Group performance status*, ICD-10* International Statistical Classification of Disease and Related Health Problems, 10th Revision*, ICD‑O* International Classification of Disease for Oncology*, OPS* German Procedure Classification

When using cancer registry data, it is important to keep in mind that the oBDS is a reporting dataset. As a rule, not only one but many institutions report information relevant to them to the cancer registry in accordance with defined reporting events. Only in the cancer registry are the individual reports combined into a data record, with multiple reports on the same case being merged into a “best information” record according to standardized rules. This combined “best-of” data record represents the actual evaluation data record that is usually required for research projects.

### Processing of cancer registry data

The state cancer registries first receive individual digital reports from medical institutions (transmitted in oBDS format). The decisive factor for transmission is fulfilment of the relevant reporting reason in the respective institution. In the case of radiotherapy (RT) reports, this is the start or end of RT. Even after being assigned to a person or tumor in the cancer registry, the individual reports cannot be evaluated immediately. The data preparation process is referred to as “best-of” because it attempts to summarize the best information (from at least two reports) about a clinical event. A special feature of the best-of for radiation therapy is the distinction between radiation therapy as the total treatment period and the actual radiation. According to this idea, radiation therapy can comprise several individual radiation treatments (referred to as “radiation”). The best-of process can be further divided into two sub-processes:

1. *Event assignment*: Matching rules are used to determine whether several reports describe the same clinical event. This sub-process determines the number of events generated from a set of messages.
2. *Feature best-of*: If the matching rules are fulfilled, competing information from the various messages is summarized based on the rules.

A common situation occurs when several institutions report the same event. For example, the start of radiation therapy may be reported both by the radiation oncologist performing the respective treatment and by the institution where the treatment was indicated. To check whether both reports describe the same event, the respective therapy date, application type, target area, and side of the target area of both reports are taken into account. Possible outcomes of this process are that both reports are created as one radiation therapy session, two radiation therapy sessions, or two different radiation therapies. When summarizing the information from both reports (feature best-of), the report with the higher information quality applies. The individual rules for event assignment and feature best-of are developed in a cross-cancer registry working group (*AG Bildung klinisches Best-of*) at the level of the § 65c platform. The aim is to achieve a standardized best-of formation in all state cancer registries. With regard to radiation therapy, the resulting evaluation dataset includes all fields listed in Table 1 under the section “Radiation therapy.” The best-of datasets created in the individual state cancer registries are made available to the ZfKD annually in a standardized data format (oBDS-RKI) and merged there into a nationwide dataset. With regard to radiation therapy, the oBDS-RKI dataset is based on the oBDS dataset, but certain data fields are not available in previous federal regulations (including information on whether boost radiation was used).

## Results

### Completeness and comprehensiveness of cancer registry data on radiation therapy

Two fundamental quality criteria for registry data are the completeness and comprehensiveness of the reports. Completeness refers to the proportion of reporting events that were actually reported to the relevant registry. In epidemiological cancer registration, there are expected values for new cancer cases that can be calculated from the data of so-called reference registries or regions. These registries are assumed to have complete coverage of all cases, and models can therefore be used to derive the expected values for other regions [11]. For the reporting events added by the clinical cancer registry in accordance with Section 65c of the Fifth Social Security Code, there is not yet a methodology for such expected values. The completeness of the registry data refers to the presence of certain information in reports that are already available to the registries. Certain essential items from the oBDS are often used to assess completeness, such as the presence of a complete TNM classification [8].

In order to assess the completeness of radiation therapy information for all German cancer registries, the dataset compiled by the ZfKD of the Robert Koch Institute (RKI) was used, which, since 2023, has also included clinical reporting events. To estimate the data completeness of radiotherapy, we identified radiation therapy recorded for each tumor case in each state cancer registry. Cases from the years of diagnosis between 2020 and 2022 from the ZfKD dataset functioned as a reference for evaluation. So-called DCO (death certificate only) cases, in which a new case is known to the cancer registry exclusively through a death certificate, and cases of non-melanocytic skin cancer (ICD-10 code C44) were not included. The dataset comprised a total of 1,735,023 tumors. There were 352,830 RT treatments for these tumors, corresponding to 0.23 RT treatments per tumor. These RT treatments included 409,666 distinct irradiations (defined as part of the *irradiation* level, e.g., with multiple target areas, possibly using different techniques), corresponding to 1.16 irradiations per reported RT treatment (range 1.00–1.45). For six federal states, the number of irradiations and therapies was identical. In these states, work is currently underway to generate the best-of list using software support, and until then, only the primary radiation therapy will be reported.

To ensure the completeness of the RT data in the ZfKD dataset, the characteristics “status in relation to surgery,” “intention of radiotherapy,” and “type of application” were used at the RT level (Table 2). The position of RT in relation to surgery was missing in 15.1% of all reported therapies. This information was completely missing in the data from four federal states. The proportion of missing data in the remaining states ranged between 0% and 5.2%. The intention of RT was missing in 0.9% of RT cases nationwide. At the state level, the proportion of missing values ranged between 0% and 4.7%. The type of radiation application was completely missing from the data of six federal states, while in the remaining ten federal states there were almost no missing values. The total proportion of missing application types was 31.0% of radiation therapies.

**Table 2:** Relative proportion of missing data on RT in the nationwide cancer registry dataset of the ZfKD at the RKI. Diagnosis years: 2020–2022

	Proportion of missing data (in %)						
	Radiotherapy level			Single irradiation level			
Federal state (pseud.)	Relation to surgery	Intention	Type of application^1^	Target side	Target area	Percutaneous^2^: chemoradiotherapy/stereotactic/breath-triggered	Brachytherapy^2^: rate type
1	0.31	0.00	100.00	–	–	–	–
2	100.00	0.56	100.00	–	–	–	–
3	0.00	0.00	100.00	–	–	–	–
4	100.00	0.00	100.00	–	–	–	–
5	100.00	0.02	100.00	–	–	–	–
6	100.00	0.03	100.00	–	–	–	–
7	0.02	0.00	0.00	0.04	0.00	7.84	6.78
8	0.00	0.07	0.00	0.04	0.07	3.11	0.00
9	0.00	0.59	0.00	0.00	0.00	42.18	31.14
10	0.00	0.02	0.00	0.03	0.04	3.37	0.00
11	5.24	4.71	0.00	1.40	4.52	36.44	0.00
12	0.02	0.00	0.00	0.01	0.01	0.09	0.00
13	1.01	0.15	0.00	0.01	0.01	7.16	0.00
14	2.04	1.36	0.04	0.36	0.38	26.78	0.00
15	0.00	0.02	0.00	0.15	0.15	14.82	0.00
16	0.08	0.03	0.00	0.11	0.19	11.44	0.00
*Overall*	*15.08*	*0.89*	*30.99*	*0.36*	*1.07*	*22.68*	*8.00*

Death certificate only (DCO) cases and cases of non-melanotic skin cancer (ICD-10 C44) were excluded from the analysis.

^1^For federal states 1–6, no data on the type of application was available in the currently available ZfKD dataset

^2^For the fields on single-session RT, only treatments with an existing type of application were selected as a reference value, and for the fields on percutaneous RT and contact RT, only treatments with the appropriate type of application

For radiation treatments, only those treatments for which a type of application was available were taken into account as a reference value for the proportion of missing information. For characteristics relating to specific types of application (chemoradiotherapy, stereotactic, breath-triggered in percutaneous RT, and rate type in brachytherapy), only the corresponding radiation treatments were considered. The information on the side of the target area was missing in 0.4% of radiation treatments (range: 0%–1.4%). The target area itself was not specified in 1.1% of radiation treatments (range: 0%–4.5%). The variables chemoradiotherapy, stereotactic, and breath-triggered were evaluated together. In 22.7% of the radiation treatments, none of this information was available (range: 0.1%–42.2%). In brachytherapy, information on the rate type (high/low/pulsed dose rate) was missing in 8.0% of cases, with a range between 0% and 31.1%.

### Research opportunities for scientific projects in radiation oncology

Interest in research using cancer registry data, as measured by the number of published articles [3] is increasing. However, medical data on radiation therapy are rarely included in these research projects.

Previous publications on radiation oncology have used other sources, including clinical cancer registries of the treating institutions with reference to patients from the corresponding catchment area, nationwide hospital billing databases, the GENESIS database of the Federal Statistical Office, data from bicentric German Comprehensive Cancer Centers (CCC), and others [12].

Reasons for this include the fact that the scope, accessibility, and potential of the data collected nationwide are unknown to those interested in research. Reports on radiation therapy are also among the more complex report formats that German cancer registries deal with. Particularly challenging is the consolidation of individual, often contradictory information into a best-of dataset. Although the cancer registries of the individual federal states consult and decide jointly on questions of best-of formation, the implementation of this (Table 2) still appears to vary. As a result, the completeness and comprehensiveness of the data currently vary between the individual federal states.

Regardless of whether a research project aggregates cancer registry data or requires it at the individual case level, the basis for this is the oBDS and the variables it includes (Table 1). The completeness and comprehensiveness of this data are important for answering clinical research questions. The state cancer registries are currently confronted with item- and entity-dependent reports with quite high missing data rates. For example, in the data from Saxony-Anhalt, information on the performance status of patients (ECOG or Karnofsky performance score) is missing in approximately 60% of surgically treated cases diagnosed with malignant neoplasms (ICD-10 C00–C97), excluding C44. In 48% of cases, information on side effects is missing for reported radiation therapy and in 81% of cases following reported systemic therapy. These results were not derived from the ZfKD dataset, as it does not include data on performance status or side effects.

Nevertheless, the potential of RT data for research is great, and the variables contained in the oBDS are likely to become the basis for many scientific questions in evidence-based medicine, as the data reflect everyday medical care (real-world data).

For example, observational studies on questions regarding the outcome (measured by vital status or tumor status) of patients treated with radiation therapy alone are well feasible. The same applies to endpoints after peri- or postoperative radiation therapy, whereby different types of application and radiation can be taken into account. The evaluation of different forms of treatment, such as surgery versus radiation, including long-term results, is possible in principle. Analyses of patient groups that benefit particularly from radiation therapy or analyses of the tolerability of certain types of radiation based on the frequency of treatment discontinuation or information on side effects are also conceivable. In addition, analyses of trends in the use and results of defined forms of radiation therapy, as well as of trends in the practical application of radiation therapy in Germany, can be performed. Studies on compliance with interdisciplinary tumor board recommendations or on the evaluation of previous recommendations from medical guidelines could be of particular relevance.

The cancer registries have implemented vital status matching with the reporting authorities. This means that follow-up reports on survival are available for all cancer cases, which enables survival time analyses in particular as a clinically relevant outcome (Tables 3 and 4).

**Table 3:** Overview of fundamental research opportunities with cancer registry data [3]

**Data type**	**Description**	**Data access**
Aggregated data	Case numbers in defined groups, e.g., by diagnosis, age, sex, region, and year	Online offers of the KR and the ZfKD or informal request
Individual case data	Individual cases, according to defined inclusion and exclusion criteria with defined variable sets	Standardized short application (form) to KR or ZfKD
Contact cancer patients	Contacting (living) patients for recruitment for studies	Application to KR with study protocol; information and consent material; vote of ethics committee; and, if applicable, data protection
Cohort matching	Comparison of a research cohort (usually with consent) to determine whether cohort members have cancer or have died from cancer	Application to KR with study protocol; information and consent material; vote of ethics committee and; if applicable, data protection
*Special cases*		
**Types of usage**	**Description**	**Data access**
Provision of data for reporting institutions	Return of complete information on reported tumor cases (including follow-up and death)	Regulated by law, on request
Comparison of mammography screening (MS)	Comparison of participants in MS to identify interval carcinomas in the MS program	Regulated by law at the state level
Comparison of colorectal cancer and cervical screening	Comparison with health insurance data by a trusted third party in accordance with § 299 of the German Social Security Code, Book V	Regulated by law at the federal and state level

*KR* state cancer registry, *ZfKD* German Center for Cancer Registry

**Table 4:** Overview of radiation oncology studies using cancer registry data

Study title	Year	Key results
Integrated prostate cancer centers might cause an overutilization of radiotherapy for low-risk prostate cancer: A comparison of treatment trends in the United States and Germany from 2004 to 2011 [13]	2015	In the USA, RT was used significantly more often in patients low-risk prostate cancer (38.4%) compared to in Germany (11.8%)
		In Germany, radical prostatectomy was performed more frequently (66.2% in Germany vs. 36.1% in the USA)
		The proportion of patients not receiving active treatment (NAT) increased in the USA from 18% to 33.2% (*p* < 0.001) but remained stable in Germany until 2009
		From 2009, the proportion of the NAT group also increased in Germany (from 15.2% to 19.4%; *p* = 0.001)
Effect of Radiotherapy in Addition to Surgery in Early Stage Endometrial Cancer: A Population-Based Study [14]	2020	RT showed positive effects in FIGO IB endometrial cancer cases (HR = 0.74) and for all histopathological grades
		However, there was no significant benefit of RT for FIGO IA cases (HR = 0.93)
		The risk of a poorer outcome in terms of survival was increased in FIGO IB cases (HR = 1.51)
Adjuvant Therapy for Elderly Breast Cancer Patients after Breast-Conserving Surgery: Outcomes in Real World Practice [15]	2023	Adjuvant RT after breast-conserving surgery improved locoregional tumor control in older patients (10-year LRFS: 94.0% with RT vs. 75.1% without RT)
		The positive effect of RT was also evident in patients with additional ET (10-year LRFS: 94.8% with RT + ET vs. 78.1% with ET alone)
Treatment patterns in stage III non-small-cell lung cancer patients: a population-based study using German cancer registry data [10]	2023	Patients diagnosed and treated in the “modern” era (2015–2018) had a significantly better survival rate (HR = 0.78; 95% CI: 0.74–0.83) compared to the period of low availability of modern diagnostics and therapies (2007–2010)
		The greatest survival advantage was seen in patients treated with RT and chemotherapy (HR = 0.73; 95% CI: 0.66–0.82)

*ET* endocrine therapy*, FIGO* International Federation of Gynecology and Obstetrics*, HR* hazard ratio*, LRFS* local recurrence-free survival, *RT* radiotherapy

### Use of cancer registries in radiation therapy: challenges and methods

Cancer registry data, as real-world data, are ideal for clinical care research; however, their use requires careful consideration of a wide range of factors [10, 19].

Incomplete datasets can lead to distortions, for example due to higher completeness in advanced tumors. It is therefore essential to understand the data and their limitations. Missing values are rarely distributed randomly (“missing at random”). and if they correlate with demographic or clinical parameters, this can distort estimates. Multiple imputation methods are helpful when data loss is limited (< 20%).

Unlike randomized controlled trials (RCTs), studies using cancer registry data must take into account differences in demographics and clinical characteristics between comparison groups, otherwise there is a risk of confounding bias. For example, patients in certified centers may have different characteristics than those in non-certified facilities, which can influence the center effect [20].

A valid evaluation requires that information about a potential confounder be available in the evaluation data, which is not always the case due to legal requirements. The future possibility of linking data to health insurance data, among other things, within the framework of the Health Data Protection Act (GDNG), could reduce this deficit in the foreseeable future.

Various biometric methods are available to reduce structural inequalities, such as multivariate models, propensity score matching, or inverse probability weighting. A clear understanding of the underlying causal relationships between the variables is essential for applying these methods appropriately. Ill-considered adjustment strategies can introduce or increase bias rather than reduce it. Directed acyclic graphs (DAGs) provide a visual and transparent framework that helps to guide variable selection and clarify the assumed causal structure.

It is also essential to define the inclusion and exclusion criteria precisely, with prospective studies providing initial guidance here. The creation of a study protocol similar to that for an RCT is referred to as emulation of a target trial. An emulated target trial aims to transfer the methodology of an RCT to the analysis of retrospective observational data by replicating a hypothetical randomized study [21]. This approach is considered good practice as it helps researchers to better anticipate bias [22].

Since radiation therapy typically does not begin on the day of diagnosis, immortal time bias is a particular concern. If follow-up begins before treatment initiation, patients who eventually receive RT are effectively “immortal” during that period, since they must survive long enough to receive the planned intervention. If not properly accounted for, this can lead to an overestimation of the survival benefit associated with treatment. To avoid immortal time bias, it is crucial to correctly align the timing of exposure and outcome. Specifically, treatment should be modeled as a time-dependent variable, such that patients are classified as exposed only from the actual start of therapy. This approach ensures that the period prior to treatment initiation is accurately attributed to the unexposed group, thereby preventing misclassification and minimizing bias in survival estimates.

In principle, delays in data transmission and processing must also be considered, which make real-time analyses with cancer registry data practically impossible [23, 24].

A further limitation concerns the evaluation of data completeness. While counting the number of radiotherapies recorded in registry data can provide an indication of coverage, there is currently no universally accepted gold standard available for validation. As a result, completeness can only be estimated indirectly, and conclusions must be interpreted with caution.

Furthermore, merging additional data sources, such as those from the German Medical Informatics Initiative (MII) data integration centers or the upcoming German Electronic Patient Record (ePA) and European Health Data Space (EHDS), with cancer registry data could provide significant opportunities for more comprehensive and advanced analyses. These linked datasets could help improve the depth and scope of research, offering more granular insights and enhancing the robustness of findings by integrating diverse health information.

## Conclusion

Due to their population-based approach, cancer registries fulfill an important function for clinical and epidemiological research in Germany. Comprehensive data on cancer diagnoses, treatments, and progression over long observation periods offer great potential for oncological health services research, which should be exploited more fully in the future [10, 18, 19].

Cancer registry data offer a previously underutilized opportunity for radio-oncological health services research, particularly in the field of radiation therapy. However, their use is associated with methodological challenges. In order to obtain reliable scientific findings, robust study designs are required that quantify and address potential biases. Careful planning with detailed study protocols in accordance with good scientific practice is essential.

Despite certain limitations, cancer registry data provide the possibility of using extensive existing datasets for specific scientific questions through innovative methodological approaches such as the emulation of a target study. Especially in radiation therapy, which is often embedded in interdisciplinary treatment strategies, cancer registry data can help to better understand treatments and their association with clinical endpoints.

In addition to their role as an independent data source, cancer registry data can be combined with other data sources, such as health insurance data, under the appropriate legal framework. The planned project related linking of such datasets will significantly expand the analysis options for health services research in radiation therapy. This will allow previously missing variables, such as comorbidities or specific therapeutic approaches, to be included in scientific studies.

Given the central importance of radiation therapy as a component of multimodal cancer treatment, the continuous improvement and integration of cancer registry data opens up new perspectives for clinical medicine. This enables more precise evaluation of existing treatment strategies and supports the further development and clinical evaluation of innovative therapeutic approaches.

## Data Availability

Aggregated result data from the cancer registry can be made available upon request, subject to applicable legal and privacy restrictions. Please contact the corresponding author for further details.

## Abbreviations

ADT: Working Group of German Tumor Centers
CCC: German Comprehensive Cancer Centers
CTCAE: Common Terminology Criteria for Adverse Events
DAG: Directed acyclic graph
DCO: Death certificate only
DKR: German Cancer Registry
ECOG: Eastern Cooperative Oncology Group performance status
ET: Endocrine therapy
FIGO: International Federation of Gynecology and Obstetrics
GDNG: Health Data Protection Act
HR: Hazard ratio
ICD-10: International Classification of Diseases, 10th Revision
ICD‑O: International Classification of Diseases for Oncology
KR: State cancer registry
LRFS: Local recurrence-free survival
MS: Mammography screening
NAT: Not receiving active treatment
oBDS: Nationwide basic oncology dataset
oBDS-RKI: Standardized oncological core dataset format used for nationwide cancer data reporting to the ZfKD
OPS: German procedure classification
RCT: Randomized controlled trial
RKI: Robert Koch Institute
RT: Radiotherapy
SGB V: German Social Code, Book V
ZfKD: German Center for Cancer Registry Data
